# The Influence of Different Sera on the Anti-Infective Properties of Silver Nitrate in Biopolymer Coatings

**DOI:** 10.3390/polym16131862

**Published:** 2024-06-29

**Authors:** Melanie Nonhoff, Jan Puetzler, Julian Hasselmann, Manfred Fobker, Silke Niemann, Georg Gosheger, Martin Schulze

**Affiliations:** 1 Department of General Orthopedics and Tumor Orthopedics, Muenster University Hospital, 48149 Münster, Germany; 2 Materials Engineering Laboratory, Department of Mechanical Engineering, University of Applied Sciences Muenster, 48565 Steinfurt, Germany; 3 Central Laboratory, Muenster University Hospital, Albert-Schweitzer-Campus 1, 48149 Münster, Germany; 4 Institute of Medical Microbiology, Muenster University Hospital, 48149 Münster, Germany

**Keywords:** biopolymer coatings, silver nitrate, periprosthetic joint infections, antimicrobial efficacy

## Abstract

The widespread prevalence of periprosthetic joint infections (PJIs) poses significant challenges in orthopedic surgeries, with pathogens such as *Staphylococcus epidermidis* being particularly problematic due to their capability to form biofilms on implants. This study investigates the efficacy of an innovative silver nitrate-embedded poly-L-lactide biopolymer coating designed to prevent such infections. The methods involved applying varying concentrations of silver nitrate to in vitro setups and recording the resultant bacterial growth inhibition across different serum environments, including human serum and various animal sera. Results highlighted a consistent and significant inhibition of *S. epidermidis* growth at all tested concentrations in each type of serum without adverse interactions with serum proteins, which commonly compromise antimicrobial efficacy. This study concludes that the silver nitrate-embedded biopolymer coating exhibits potent antibacterial properties and has potential for use in clinical settings to reduce the incidence of PJIs. Furthermore, the findings underscore the importance of considering serum interactions in the design and testing of antimicrobial implants to ensure their effectiveness in actual use scenarios. These promising results pave the way for further research to validate and refine this technology for clinical application, focusing on optimizing silver ion release and assessing biocompatibility in vivo.

## 1. Introduction

The field of biopolymer implant coatings has emerged as a promising solution to combat implant-related infections, which are particularly prevalent in orthopedic surgery [1]. These infections pose a significant challenge, with mega-endoprostheses carrying a substantial risk of periprosthetic joint infection (PJI), reaching approximately 20% and escalating to over 50% after multiple revisions [2,3,4,5]. The primary pathogens responsible for PJIs are *Staphylococcus aureus* and coagulase-negative staphylococci, notably *Staphylococcus epidermidis* [6].

To combat this, an implant coating comprising silver nitrate embedded within the biopolymer poly-L-lactide has been developed. The silver nitrate is contained within a reservoir in the polymer, facilitating the controlled release of silver ions exclusively at the material surface. This reservoir serves a dual purpose: safeguarding surrounding cells from cytotoxicity and enabling selective activation through non-invasive shock waves upon the occurrence of bacterial infection. A comprehensive assessment of the coating’s efficacy has been conducted through a mechanical and microbiological testing concept [7]. The results demonstrate a significant inhibition of biofilm formation and the antimicrobial properties of the shock wave-induced silver release mechanism [8,9]. Focused high-energy shock wave therapy is a non-invasive clinical therapy that is employed in orthopedics for the treatment of conditions such as pseudoarthrosis, tendonitis, enthesopathies, and slow fracture healing [10,11]. The shock waves are acoustic waves that traverse the soft tissue and release their energy due to an impedance change at hard surfaces, such as bone or titanium implants. With regard to the coating, the shock waves locally detach the biopolymer in small areas, thereby facilitating a burst release of silver from these areas.

In the past, a discrepancy between in vitro and in vivo studies has been observed in numerous investigations utilizing silver as an antibacterial agent [12]. This discrepancy is also evident within different in vitro assays due to the use of varying culture media for bacterial growth and their interactions with silver ions [12,13]. Consequently, the conventional methodology of the zone of inhibition test for silver is rendered inapplicable due to these interactions, necessitating the adoption of alternative assays to measure antibacterial efficacy [13]. Hidalgo et al. (1998) observed diminished efficacy of silver nitrate in the presence of fetal bovine serum (FBS), and moreover, bovine serum albumin was found to attenuate the impact of silver nanoparticles on various bacterial strains, including *S. aureus*, *Streptococcus salivarius*, *Escherichia coli*, and *Pseudomonas aeruginosa*, within an agar matrix [14,15]. Similarly, Liau et al. (1997) noted the neutralization of silver nitrate by glutathione (GSH) due to its cysteine component [16]. Cysteines are characterized by thiol functional groups, which serve as sites for the binding of silver ions. They are also present in albumin, the predominant protein component in blood serum. The interaction between GSH and albumin with silver ions leads to an elevation in the minimum inhibitory concentration (MIC) of silver nitrate when these proteins are present [17]. The binding of silver ions to thiol groups additionally represents a mechanism contributing to the antibacterial properties of these ions. The ions inactivate coenzyme A, a pivotal enzyme involved in the tricarboxylic acid (TCA) cycle, which possesses thiol groups. Through the inactivation of this enzyme, the normal cellular respiration of bacteria is disrupted, ultimately resulting in bactericidal effects [18].

The differences between the physiological fluids of various species have been documented for several decades. In 1945, Moore published a study on the differing electrophoretic patterns observed in sera from different species and their associated proteins [19]. Warren et al. (2010) investigated macrophage stimulation in the blood of different species and found that, in particular, mice and humans exhibited a considerable difference in the induction of cytokines by serum proteins [20]. The compounds formed by nanoparticles and serum proteins also vary greatly depending on the species, as evidenced by a comparison between human serum, FBS, and mouse serum with gold and silica nanoparticles [21]. The differences between the serum albumins in different species are particularly well known. There are differences in enzymatic, transport, redox, and binding activity, as well as structure, that influence the behavior of the albumin used in diagnostics and other applications [22,23,24]. For example, bovine serum albumin and human serum albumin share only 76% of their identity [23,25].

The evaluation of a medical device and/or drug comprises several phases, including in vitro testing, preclinical in vivo studies, and clinical studies. In the in vitro tests, FBS is utilized as a standard for cell culture. For the preclinical studies, an in vivo model must be selected. Small animals, most commonly mice, are often employed for this purpose. The choice of the animal model is often based on the cost and size of the animal. However, if there are potential limitations, such as differences in serum composition and thus differences in efficacy, these should be considered when selecting the model. In the case of clinical studies and, of course, later clinical application, the influence of the human body and thus of the human serum must also be contemplated.

The primary aim of this investigation is to evaluate the degree to which the antibacterial effectiveness of released silver diminishes when silver ions associate with thiol groups, especially within GSH and albumin. The findings from this study provide an evaluation of the antibacterial performance of silver released from a biopolymer coating in different evaluation phases and across multiple biological systems.

## 2. Materials and Methods

This study was designed to investigate the impact of sera from four distinct sources on silver nitrate. To achieve this, a series of growth curves were recorded, focusing on the potential variations in protein compositions and their consequent impacts on ion binding.

**Figure 1:**
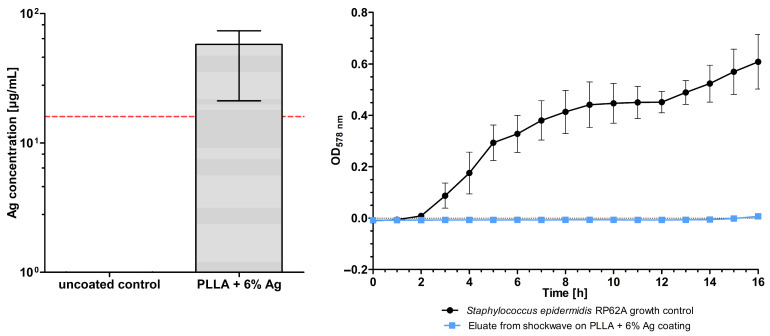
(**Left**) Silver release by shock waves on poly-L-lactic acid (PLLA) and 6% silver coating compared to uncoated samples. The red line indicates the minimal inhibitory concentration of *Staphylococcus epidermidis* RP62A. (**Right**) Inhibition of *S. epidermidis* growth through eluate from shock wave on poly-L-lactic acid and 6% silver coating. All data are from Puetzler et al. (2023) [8].

Four different sera were intended to map the complete evaluative trajectory of the coating across several experimental contexts, encompassing in situ clinical scenarios, in vivo, and in vitro, and to reveal any possible influences during every step of the validation process of the coating. For the simulation of clinical applications, human serum was utilized; FBS was employed for the in vitro experiments; and both mouse and rabbit sera were used for the in vivo assessments. Leukocyte-depleted frozen fresh plasma was anonymized and distributed by the blood bank of the University Hospital Muenster for research purposes. The plasma was thawed and left to coagulate before being centrifuged at 3000 rpm for 10 min to retrieve the serum. FBS, rabbit serum, and mouse serum were purchased from PAN-Biotech GmbH (Aidenbach, Germany). Test solutions were created using silver nitrate (Carl Roth GmbH + Co. KG, Karlsruhe, Germany) in aqua dest. stock solution and either serum or tryptic soy broth (TSB; Becton Dickinson GmbH, Heidelberg, Germany) samples. This step was performed first to maximize the binding effect of the silver ions to any components of the serum and to ensure that bacteria could only be inhibited subsequently. In a previous study, it was determined that the average shock wave-induced release of silver from the 6% silver coating was 57.8 mg/L [8]. From this study, it is known that the concentration effectively inhibits bacterial growth (Figure 1) [8]. Using this value, the concentrations of 50, 100, 150, and 200 mg/L of silver nitrate were selected for these solutions. Only the two lower concentrations were used for the TSB controls.

*S. epidermidis* RP62A (ATCC-35984; American Type Culture Collection, Manassas, VA, USA) was cultured in TSB overnight at 37 °C with orbital shaking. This strain was chosen for its capacity to form high-quality biofilms and has been utilized in the majority of prior assessments of the coating’s efficacy. The overnight culture was then adjusted to an optical density of 0.010 at 578 nm and then diluted 1:10 with TSB, resulting in a bacterial count of approximately 5 × 10^5^ colony-forming units/mL (CFU/mL).

The test solutions were added to the wells of a 96-well plate as technical duplicates and biological triplicates, each consisting of 50 µL. Additionally, 50 µL of the adjusted inoculum was added to each well, and the same amount was pipetted as a growth control in technical duplicates and biological triplicates. Each test solution was accompanied by a separate blank control consisting of 50 µL of the test solution and 50 µL of TSB in technical duplicates to allow for later blank correction after the measurement.

The bacterial growth in the solutions was monitored using optical density. The measurement was performed with the Synergy HTX Multi-Mode Reader (BioTek Instruments GmbH, Bad Friedrichshall, Germany) at 578 nm, 37 °C, and orbital shake with a speed of 282 cpm every 30 min for 16 h. After the measurement, the data were blank-corrected in Microsoft Excel (Microsoft Corporation, Redmond, WA, USA) and graphically analyzed in GraphPad Prism 5 (GraphPad Software Inc., Boston, MA, USA).

## 3. Results

To assess the effect of silver released from a polymer coating in situ, bacterial growth curves were utilized. A minimum concentration of 57.8 mg/L was assumed based on a previous investigation of the active shock wave release of silver from the coating [8]. This concentration could potentially be increased by adjusting the handling and/or increasing the number of areas being activated. Four concentrations of silver nitrate were selected for testing to reflect this. Thus, the two lower concentrations were also utilized in TSB as a control. The optical density measurement, after being corrected for the blank, indicates the concentration of bacteria in the solution.

**Figure 2:**
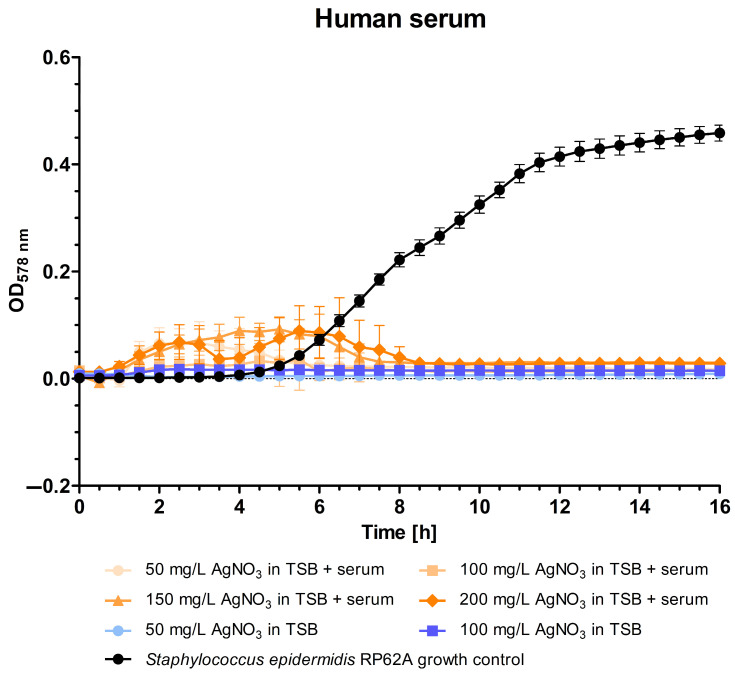
Optical density (OD) at 578 nm of solutions containing different concentrations (50, 100, 150, and 200 mg/L) of silver nitrate in human serum and tryptic soy broth over a 16 h period. The optical density corresponds to the growth of *Staphylococcus epidermidis* RP62A.

The silver samples demonstrated no growth in either human serum or TSB compared to the control curve with *S. epidermidis* RP62A (Figure 2). The control grew to a mean optical density of 0.459. In contrast, the curves from the TSB samples remained largely unchanged, with a maximum at 0.016. This indicates that silver nitrate at concentrations of 50 and 100 mg/L in TSB inhibits the growth of the bacteria. At 16 h, the maximum optical density of all the silver nitrate in human serum samples was 0.034, which demonstrated growth inhibition. However, there were fluctuations in the optical density in the first eight to nine hours. During this time, the variance between samples (i.e., the different wells) of a single test solution was considerable.

To examine whether there are any differences between sera of different origins, this methodology was also tested on three distinct animal sera. These sera were selected based on their involvement in different stages of the typical validation process of a medical device or pharmaceutical: in vitro and in vivo tests. FBS was selected because of its typical use in cell culture. To reflect diverse small animal models and their potential variations, both mouse and rabbit sera were chosen.

**Figure 3:**
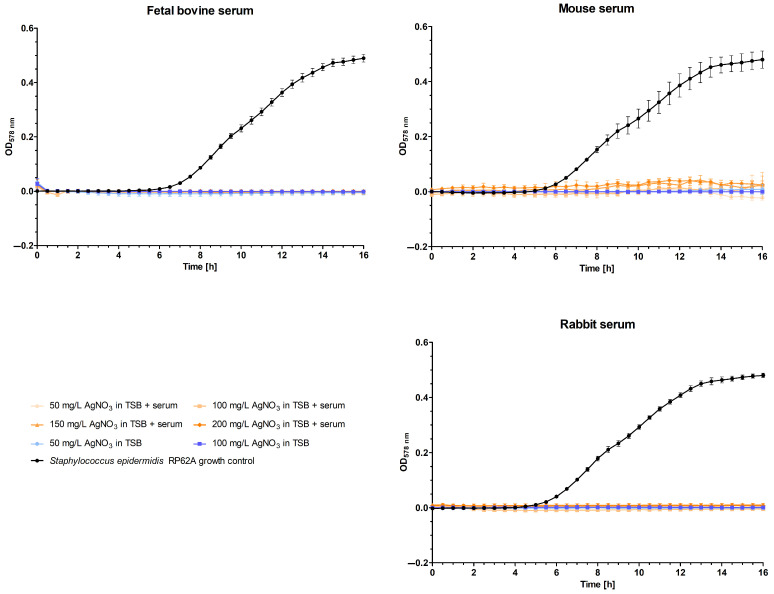
Optical density (OD) at 578 nm of solutions containing different concentrations (50, 100, 150, and 200 mg/L) of silver nitrate in various animal sera (fetal bovine, mouse, and rabbit) and tryptic soy broth over a 16 h period. The optical density corresponds to the growth of *Staphylococcus epidermidis* RP62A.

In general, no observable growth was identified in any of the animal sera (Figure 3). The maximum optical densities at 16 h observed in FBS, mouse serum, and rabbit serum were 0.025, 0.104, and 0.019, respectively.

## 4. Discussion

This study has demonstrated that silver nitrate effectively inhibits the growth of *S. epidermidis* across various serum environments, underscoring its potential utility in preventing implant-related infections in orthopedic surgeries. The consistent inhibition of bacterial growth in human serum, FBS, mouse serum, and rabbit serum, as demonstrated through controlled optical density measurements, points to the robust antibacterial properties of silver nitrate, reinforcing its value in clinical applications.

The results indicate that silver is not inhibited by the proteins in any of the sera mentioned at a concentration of 50 mg/L silver nitrate or higher. However, it is still possible that at a lower silver nitrate concentration, the binding of silver to thiol groups in 50% serum may lead to an increase in the minimum inhibitory concentration (MIC) in this medium. With 50 mg/L silver nitrate, the free binding sites are all occupied, resulting in saturation. It may be assumed that a significant proportion of the silver nitrate remains in its free form at this particular concentration, thus allowing the antimicrobial ions to exert their effect. It is assumed that a minimum release of approximately 50 mg/L of silver ions is present as a result of the shock waves produced by the biopolymer coating.

**Figure 4:**
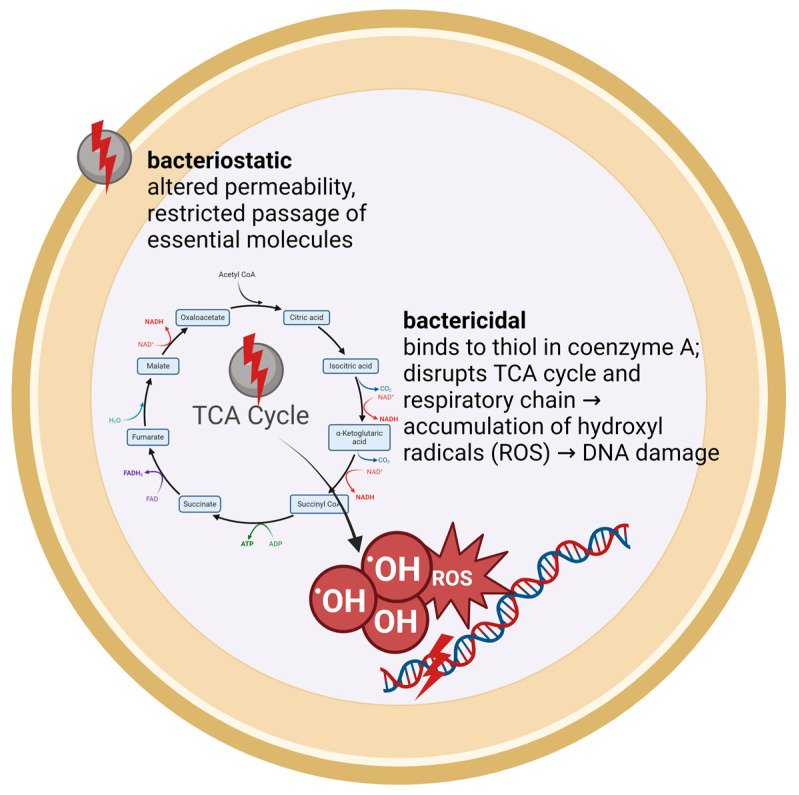
Schematic illustration of the antibacterial effect of silver ions.

Silver ions exhibit dual antimicrobial mechanisms against bacteria, which can be categorized into bacteriostatic and bactericidal effects. Initially, silver ions target the murein wall of bacterial cells, binding to it and altering its permeability. As a bacteriostatic strategy, this initial interaction prevents the proliferation of bacteria by restricting the passage of substances essential for their growth and survival. Subsequently, the bactericidal action of silver ions manifests as they bind to thiol groups present in bacterial enzymes, leading to enzyme inactivation. This binding critically impairs metabolic processes, including the TCA cycle and the respiratory chain. The disruption results in the accumulation of hydroxyl radicals, which are detrimental to bacterial DNA and further contribute to the bactericidal outcome (Figure 4) [18,26].

In a similar manner, the silver ions can bind to the thiol groups of the cysteine component of GSH and albumin. GSH and albumin act as antioxidants, protecting against free radicals and reactive oxygen species (ROS) [17]. Mulley et al. (2014) tested silver nitrate concentrations in human serum, human serum albumin, and GSH. The MIC of silver nitrate for *S. aureus* increased from 33 µmol/dm^3^ to 1121 µmol/dm^3^ when 1 mmol/dm^3^ GSH was present. The MIC was found to increase to a lesser extent in human serum and human serum albumin. The MIC in 50% human serum was determined to be 174 µmol/dm^3^, which corresponds to 29.56 mg/L silver nitrate. It should be noted that the MIC is not directly comparable to the results presented here, as different media and a different bacterial strain were used. However, the results of this study do not contradict those of Mulley et al. (2014), as the former began at a significantly higher concentration of silver nitrate [17].

The findings of this study, while promising, also highlight several challenges and opportunities for advancement, particularly with its reproducibility. Fluctuations in the first eight to nine hours of the human serum incubation period suggest the hypothesis that there are initial stages of growth observed in select wells. However, it is more probable that these fluctuations are a consequence of alterations in serum coloration resulting from incubation at body temperature or the presence of residual coagulation factors. The protein compositions of the various wells may exhibit slight variations, which could potentially influence the interactions between the proteins or their folding dynamics due to the change in temperature. This could result in subtle differences in coloration. This conclusion is based on the observed differences between the various wells and the negative values observed after blank correction. Given that the serum was produced manually from fresh plasma of uncontrolled origin, this explanation seems like a plausible hypothesis.

The clinical translation of these results could significantly impact the management of PJI, potentially lowering infection rates associated with orthopedic implants. The translation from the laboratory bench to the bedside involves not only confirming these findings in clinically mimetic models but also considering physiological factors such as the investigated interactions between serum proteins and the active agent that might influence efficacy in human patients.

Overall, while these in vitro assessments provide valuable insights into the interactions between silver ions and different sera, they also prompt a reevaluation of the typical experimental designs and models used in preclinical testing. Enhancing the predictiveness of these models could accelerate the development of antimicrobial coatings that are both effective and clinically viable, ultimately reducing PJI rates and improving patient outcomes. Possible alterations could be to use physiological fluids as well as a co-cultivation of cell lines and bacteria, mimicking the race for the surface of the implant [27].

The promising results from this study pave the way for several key future research directions that are essential for advancing the clinical application of silver-based antimicrobial coatings. While determining the MIC in sera could be informative, it may not be directly relevant to the current focus, which instead lies in optimizing the therapeutic window of silver release. Enhancing this aspect might involve testing different intensities or frequencies of the shock wave therapy used to trigger silver ion release, thereby ensuring consistent antimicrobial activity while avoiding adverse effects on surrounding tissues. Furthermore, in vivo studies are needed to monitor their overall safety, followed by clinical trials to test their practical viability. The insights gained from such investigations will not only validate the effectiveness of silver-based coatings in actual medical settings but will also refine their application protocols to maximize patient benefits and minimize risks.

## 5. Conclusions

In summary, the research presented in this paper demonstrates the efficacy of a biopolymer implant coating embedded with silver nitrate in inhibiting bacterial growth, particularly that of *S. epidermidis*, across a variety of serum environments. This study effectively highlights the potential of this approach for mitigating implant-related infections in orthopedic settings. The results also demonstrate that the effect of silver nitrate in the new polylactide coating remains consistent across different environments, as the anti-infective efficacy does not significantly diminish in various sera. This suggests that the observed in vitro effect should not be attenuated by these factors in an in vivo situation. Importantly, the findings also underscore the complex interactions between silver ions and serum proteins, which could influence the clinical translation of this technology, thereby guiding future research towards optimizing the conditions for clinical application to improve patient outcomes in the management of PJIs.

## 6. Patents

A patent application has been filed for the coating (international publication number: WO2023025944).

## Data Availability

The data presented in this study are available upon request from the corresponding author. The data are not publicly available due to privacy restrictions.
